# A Contribution to the Treatment of Hay Fever

**Published:** 1910-07

**Authors:** George A. Leitner

**Affiliations:** Piermont, N. Y.; Surgeon Nyack Hospital; Attending Physician St. Agnes’ Orphan Asylum, Sparkill, N. Y., etc.


### ABSTRACT

### A Contribution to the Treatment of Hay Fever

By GEORGE A. LEITNER, M.D., Piermont, N. Y.
Surgeon Nyack Hospital; Attending Physician St. Agnes' Orphan Asylum, Sparkill, N. Y., etc.

*American Medicine*, May, 1910.

ALTHOUGH much has been written on the subject of hay fever and numerous methods have been suggested for its treatment, none of these can be regarded in the light of specifics. This is not at all surprising when it is considered that in hay fever we are not dealing with a distinct pathological entity, but rather with a complex of symptoms having many sources of origin.

Much more is known concerning the exciting than the predisposing causes, the former comprising chiefly the pollen of various plants—in this country particularly ragweed and golden rod—as well as dust and animal emanations. As regards the predisposing factors these are to be sought first in pathological conditions in the nasal cavities, such as the presence of spurs and deflections of the septum, hypertrophied turbinates, inflammation of the antrum, and also in a neurotic disposition.

In view of the diversity of factors concerned in the etiology of hay fever it is apparent that each case is a law unto itself and must be treated individually, ascertaining if possible the underlying cause. In a general way the cases may be grouped into those in which distinct pathological changes are found in the nose, and those in which the hyperesthetic condition of the nasal mucous membrane is but a part of a general neurosis.

In both of these classes of cases the results of treatment, short of a change of climate, still continue far from satisfactory. When, therefore, my attention was called to the possible value of a special culture of the bacillus of Massol in this condition, through an article by Dr. H. Holbrook Curtis (*Merck's Archives*, January, 1909) I decided to give it a trial. Dr. Curtis in this article stated that the active solution of this culture had a pronounced effect upon the vasomotor system of the nose, so that turgescent hypertrophies seemed to disappear and edematous conditions resulting from hay fever subsided. It may be mentioned here that the preparation of lactic acid bacilli employed in Dr. Curtis's experiments has since been introduced under the name of massolin.

The results so far obtained have been most interesting and encouraging. I found that in those cases in which there were distinct pathological conditions in the nasal cavities, such as hypertrophic rhinitis, nasal spurs, polyps and sinus troubles, the use of this culture proved it to be almost a specific. Some of the cases were relieved immediately and others were greatly benefited. In those cases where the underlying cause was undoubtedly a neurosis there was not the slightest evidence of any benefit.

Although the removal of pathological changes in the nose when present in cases of hay fever is usually considered an essential feature of treatment, it has been found that in not a few cases the disorder nevertheless persists, thus showing that these lesions are to be considered only as contributory causes. It seems probable that in such cases the massol bacilli either directly antagonise the toxic element in pollen, or so modify existing pathological conditions as to increase the resistance power of the mucous membrane towards the morbific agent.

The series of cases under consideration were all chronic, dating back in the most recent case eight years. All had been subjected to various operations or the usual treatment with adrenalin, oils, cocaine, etc., without relief.

In most of the cases, treatment with the solution of massol bacilli was not required beyond several days and a most interesting fact was that the majority remained immune to the exciting cause without further attention. The following series of cases are illustrative:

Case I.—Sister D. had been a victim for over eight years of most pronounced hay fever symptoms in which the irritant was undoubtedly the blossom of hay. Her history had been one of most pronounced incapacitating attacks during the blossoming of hay. She had been confined to her room for some days when treatment with massolin was established, beginning on a Saturday afternoon. On Sunday morning the change in the facial expression especially in connection with the eye disturbance, was most marked and by Monday she was entirely free from all symptoms.

Case II.—Mrs. W. presented the usual train of symptoms, the exciting cause being undetermined. She had suffered for years and had submitted to various operations for the relief of this trouble. She was under the care of a conscientious and competent physician who, after trying practically all of the known remedies, as a last resort, put her on massolin applied by spray. The results in this case were immediate though it was necessary to continue treatment on and off throughout the season.

Case III.—Mrs. H., a trained nurse, had been a sufferer for many years and had undergone very extensive cleaning out of the nasal passages. At the time of commencement of treatment with massolin she was practically confined to the house and unable to attempt any professional work. Forty-eight hours' treatment established a cure that was permanent for the balance of the season.

To recapitulate, we must bear in mind that neurotic cases are not in the least benefited. Cases with pathological nasal conditions are benefited only upon proper application of the lactic bacilli solution directly to the diseased area.

As to the method of use the nasal tract should be washed out with warm normal salt solution, cleansing it thoroughly and then by means of a spray, swab or canula, massolin liberally applied to the involved mucosa, repeating the application two or three times a day till the symptoms subside.

